# Morphology of Roots and Canals of Maxillary First Premolars: A CBCT Study in a Peruvian Sample

**DOI:** 10.1155/2024/2341041

**Published:** 2024-10-01

**Authors:** Juan Salvador Yanqui-Gómez, Julissa Amparo Dulanto-Vargas, Kilder Maynor Carranza-Samanez

**Affiliations:** ^1^ School of Dentistry Universidad Científica del Sur, Lima, Peru; ^2^ Research Group in Dental Sciences School of Dentistry Universidad Científica del Sur, Lima, Peru

**Keywords:** canal configuration, cone-beam computed tomography, maxillary premolars, root morphology

## Abstract

**Introduction:** It is important to take into account variations in structures related to dental pulp for planning the most adequate endodontic treatment management. The objective of this study was to determine the morphology of roots and canals of maxillary first premolars (MFPs) using cone-beam computed tomography (CBCT).

**Materials and Methods:** This retrospective study included a sample of 392 CBCTs of Peruvian adults proportionally selected by sex, age, and quadrant. One MFP per individual was selected for evaluation by a calibrated evaluator based on the number of roots and canal configuration according to the Vertucci classification (VC; Cohen's *κ* ≥ 0.834). Pearson's *χ*^2^ and Kruskal–Wallis tests were used with a significance level of *P* < 0.05.

**Results:** Most MFP presented double roots (59.9%) and were VC type IV (52%). Morphology showed a nonsignificant difference by quadrants (*P*=0.994). A significant positive association was found between the presence of double roots and type IV in men and with older age, while single roots and type I and II were associated with women and younger age (*P* < 0.05). Double roots were associated with VC type IV (86%) and single roots with types III (34%), II (32%), and I (26%; *P* < 0.001).

**Conclusions:** MFPs in a Peruvian sample presented a higher frequency of double roots with two separate canals. The morphology of root and canals was associated with age and sex.

## 1. Introduction

Knowledge of dental anatomy is essential for performing dental treatment and anthropological identification [1]. The external root morphology often conceals variations in the canals [2], where it is common to find a complex canal configuration in permanent dentition [3]. Awareness of these variations is crucial for the long-term success of endodontics [4]. Preconception without thorough evaluation of the canals system can lead to incorrect diagnoses and problems, such as difficulty location, instrument breakage, and incomplete obturations [5, 6].

Diversity in the number and shape of roots and canals was studied in the first classifications in the 1970s [7]. These standardized methods typed human canal morphology based on the pattern of division from the pulp chamber to the root apex [8]. The Vertucci classification (VC) is still widely used. It was developed in 1984 following the evaluation of 2400 teeth based on eight configurations considering the branching of the main root canal, the location of the apical foramen, transverse anastomosis, and the frequency of the apical delta [9].

Various techniques assess pulp canals by sections, ink injection, macroscopy, and scanning electron microscopy [10], with staining being the “gold standard,” although it is not applicable in *in vivo* analysis [11]. Clinical research using imaging has advanced in accuracy and application in large samples [12]. Noninvasive radiographic techniques include 2D imaging (periapical and orthopantomography) and 3D imaging with spiral computed tomography (CT), micro-CT, and cone-beam CT (CBCT) [4]. CBCT has demonstrated to be reliable and is widely used for quantification and qualification similar to staining [13, 14].

Among previous studies [3, 15–32] evaluating the characteristics of the canals and roots of maxillary first premolars (MFPs) in adults by CBCT (Table 1), VC type IV [3, 15–18, 20–23, 25–30, 32], and double roots [3, 15, 19, 22, 23, 26–28, 30, 31] have frequently been found. However, other studies have described variations, including the similarity of single or double roots [18, 20, 21, 25, 29] or the predominance of a single root [16, 17, 24, 32] and VC type I [19, 24, 31]. Differences were attributed to factors such as ethnicity, age [21], and sex [15, 20, 21, 23, 27, 28, 30, 32].

Review of the literature to date found no studies on the internal anatomy of MFP in large samples of an adult Peruvian population. These data could be useful for the diagnosis and clinical endodontic treatment of MFP [33]. Therefore, the aim of this study was to determine the root and canal morphology of MFP in a sample of adult Peruvians using CBCT.

## 2. Materials and Methods

### 2.1. Study Design and Ethical Aspects

This retrospective, cross-sectional, and observational study was approved by the Institutional Research Ethics Committee of the Universidad Científica del Sur (No. 081-CIEI-CIENTÍFICA-2022) and was conducted following the STROBE guidelines (Table S1) and the principles of the Declaration of Helsinki.

### 2.2. Sample Selection

From the analysis of 824 CBCTs obtained from a private radiological center in Lima (Peru), from 2017 to 2021, a sample of 392 CBCT from adults aged 20–60 years (mean age: 38.72 ± 11.54 years) was formed. One MFP was randomly selected per individual until completing a proportional distribution of groups based on sex, age group (20–29 years, 30–39 years, 40–49 years, and 50–60 years), and quadrant (right or left). The inclusion criteria were radiographs of individuals with Peruvian nationality with at least one fully formed MFP root. CBCT images with distortion in the examined area and MFP with resorption, periapical lesion, calcification, or dental treatment were excluded. A minimum sample size of 385 was calculated using Epidat v.4.2 software to estimate an unknown proportion of MFP morphology types of 50% from an infinite population, with a confidence level of 95% and precision of 5%. The sample was increased to 392 CBCT to achieve homogeneous subgroups.

### 2.3. Image Acquisition

Images were obtained with a PaX-i3D Smart CBCT device (Vatech, Korea) with a configuration of 50 kV, 4 mA, voxel size of 0.2 mm, field of view 5 cm × 5 cm, slice thickness of 0.1 mm, and exposure time of 13–15 s. The Digital Imaging and Communications in Medicine (DICOM) format data were transferred to ITK-SNAP 2.4.0 software. Image analysis was conducted in a low-light environment, including a 23-inch monitor with a resolution of 1366 × 768 pixels, and adjustments in brightness, contrast, and magnification. The data were anonymized to register sex and age data later.

### 2.4. Pilot and Calibration

The principal investigator (J.S.Y.G.) was previously trained and calibrated in the number and type of root and canal morphology using the software by a dentist and researcher (K.M.C.S.). A pilot sample of 30 CBCT was used to value interexaminer and intraexaminer agreement (reexamination 2 weeks later). The sample was treated with replacement until an excellent calibration was achieved according to Landin and Koch (Cohen's *κ* ≥ 0.834) and was excluded from the study.

### 2.5. Root and Canal Morphology

**Figure 1:**
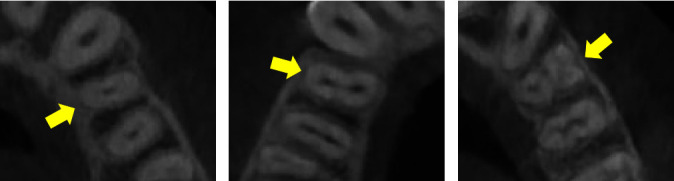
Variations in the number of roots in maxillary first premolars: (A) one root, (B) two roots, and (C) three roots.

The examiner performed up to 20 CBCT observations per day using axial, sagittal, and coronal planes. The number of roots was evaluated as follows: single root (single or bifid apical tip), double root (bifurcated roots with partial or complete separation or deep groove), and triple root (three roots with partial or complete separation or deep groove) [18–21, 23, 32]. Root morphology was assessed in terms of the number and buccal, palatal, mesial, and distal position (Figure 1).

**Figure 2:**
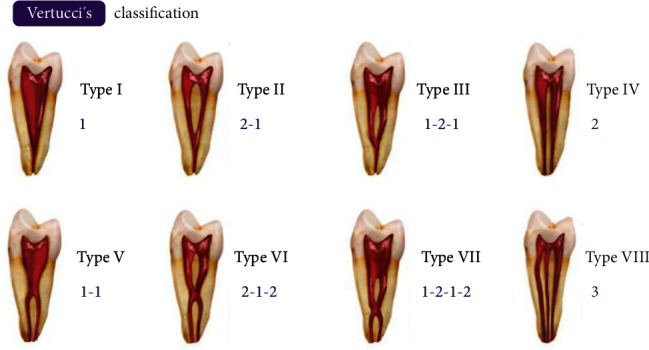
Vertucci classification of canal morphology.

Canal morphology was evaluated according to the eight variants of the VC [9]: Type I (1): a single canal from the pulp chamber to the apex; Type II (2-1): two canals leaving the pulp chamber, merging to exit as one at the apex; Type III (1-2-1): one canal dividing into two, subsequently joining to exit as one; Type IV (2): two separate canals from the chamber to the apex; Type V (1-2): one canal dividing just below the apex into two separate canals with separate apical foramina; Type VI (2-1-2): two separate canals in the pulp chamber that unite in the root and then divide again at the apex; Type VII (1-2-1-2): one canal leaving the pulp chamber, dividing and uniting in the root, and finally dividing into two distinct canals; and Type VIII (3): three separate canals from the pulp chamber to the apex. The VC image was created using Adobe Photoshop v.23.5.1 software (Figure 2).

### 2.6. Statistical Analysis

The descriptive analysis included frequencies, percentages, means, standard deviation (SD), medians, and interquartile range (IQR). Morphology was compared based on sex, age groups, and quadrant using the Pearson's *χ*^2^ test. Age (years) was not normally distributed (Kolmogorov–Smirnov) and was analyzed using the Kruskal–Wallis test with pairwise analysis for comparison of morphology. The data were analyzed using the SPSS v.26 statistical software (Statistical Package for the Social Sciences, IBM, Armonk, New York, USA) at a confidence level of 95%.

## 3. Results

The distribution of MFP according to sex, age, and quadrant characteristics is shown in Table 2.

All the MFP with double roots were of buccal and palatal types, while triple roots were located in palatal, mesial, and distal positions. The morphology of MFP roots was significantly associated with sex (*P*=0.004) and age (*P*=0.002), but not with the quadrant (*P*=0.994). Double roots were the most common (59.9%) and were associated with male sex (55.7%) and older age (42 years, IQR = 18, ≥40 years = 58.3%). Meanwhile, single roots were the second most prevalent (37%), being associated with women (60.7%) and younger age (35 years, IQR = 19, ≤39 years = 63.5%). Triple roots were infrequent (3.1%), with the prevalence being similar regardless of sex or age (Table 3).

The most frequent VC type in MFP was type IV (52%), with a lower prevalence of types III, II, I, and V (between 8.2% and 12.5% in each group), and types VIII, VII, and VI (between 0.8% and 3.1% in each group). Canal morphology was significantly related to sex (*P*=0.022), age (*P* < 0.001), and the number of roots (*P* < 0.001), but not to the quadrant (*P*=0.993). Older age was associated with VC types II (43.5 years, IQR = 11) and IV (42 years, IQR = 18, ≥60 years = 58.3%), while younger age was related to type I (31.5 years, IQR = 11, ≤39 years = 86.8%). The most prevalent canal configuration was VC type II in women (73.9%) and type IV in men (54.9%). Types I, II, III, VI, and VII were associated with single roots (100%), while types IV and V predominated in double roots (≥99.5%), and type VIII in triple roots (100%; Table 4).

**Figure 3:**
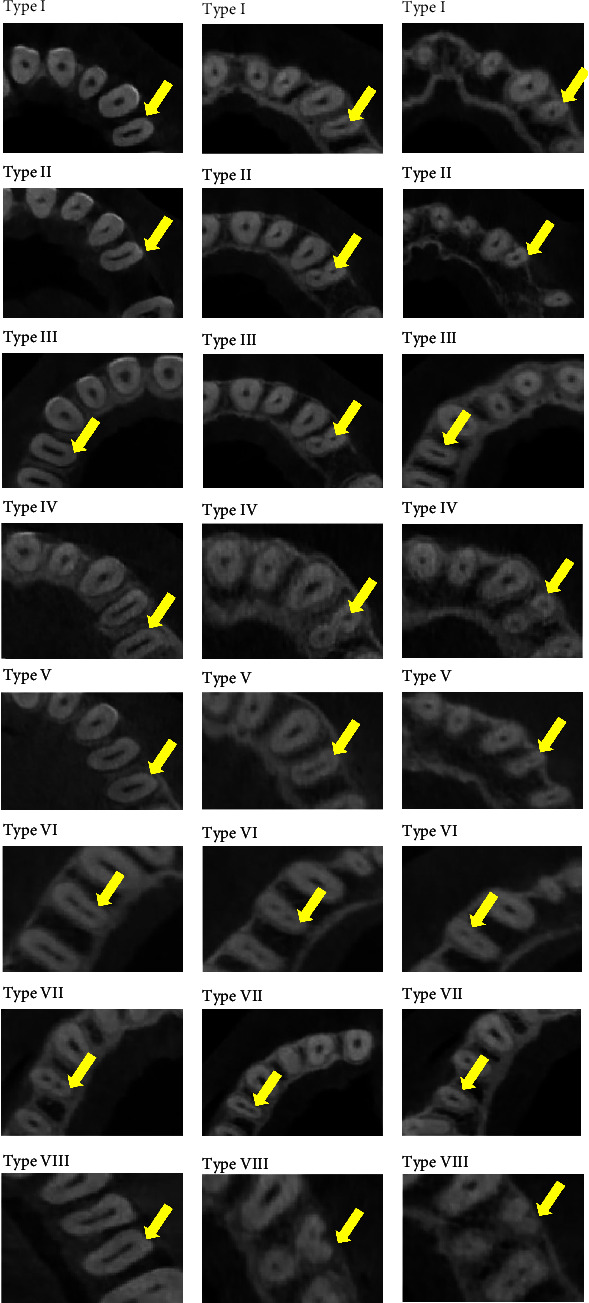
Cross-sectional views of the types of maxillary first premolars according to the Vertucci classification using CBCT. (A) Coronal, (B) middle, and (C) apical.

A selection of canal configuration in the coronal, middle, and apical sections of CBCT images is presented in Figure 3.

## 4. Discussion

MFPs present more internal root and canal variations compared to other types of premolars in which single roots and canals predominate [12]. After molars, this type of tooth is a frequent candidate for pulp disease, making knowledge of its anatomy and associated factors essential for the success of endodontic treatment [34]. This study found that the most common root morphology in MFPs in a sample of Peruvian adults aged 20–60 years was the double root with a VC type IV configuration.

Previous CBCT studies evaluated a total of 11,323 MFP from 9072 individuals aged 12–84 years in 14 subpopulations: Brazil [15], China [16, 17], India [18, 19], Iran [20], Iraq [21], Israel [22], Germany [23], Malaysia [24], Portugal [25], Saudi Arabia [3, 26, 27], South Africa [28], Spain [29], Turkey, and Cyprus [30–32]. The present study included a large sample of Peruvian adults, matched for sex and age. Half of the previous studies controlled sex [17, 23, 24, 26–28, 30–32], but few age [21]. Similar to most reports [15–25, 27, 29], the CBCT voxel size used was small (≤200) as recommended for achieving accurate anatomical images [35].

We found that double roots predominated over single roots (ratio: 1.6). This finding was also described in Brazil [15], India [19], Israel [22], Germany [23], Saudi Arabia [3, 26, 27], South Africa [28], and Turkey [30], where the frequency of double roots ranged from 54.1% to 86.2% compared to single roots, which ranged from 12.5% to 40.7% (ratio: 1.4–6.9). However, the prevalence of single roots was twofold compared to that of double roots in Asian subpopulations in China [16, 17] and Malaysia [24], and the prevalence of single or double roots was similar in India [18], Iran [20], Iraq [21], Portugal [25], Spain [29], and Turkey–Cyprus [31, 32]. On the other hand, our results and those of others have shown that the presence of three or more roots is infrequent [3, 15–32].

From the review of previous studies on canals morphology with VC [3, 15–32], the prevalences from highest to lowest were: type IV (2%–82%), I (2%–76%), II (<34%), V or III (<16%), VI (<14%), and VII or VIII (<3%). The untypified configuration was infrequent with <5% [16, 17, 21, 25, 26, 32]. In the present study, the canal system varied and presented in the lower (VC types I and VI), middle (VC types II, IV, and V), and high quartile (VC types III, VII, and VIII) of the average of the results of previous studies. In our study the prevalence of VC type IV (52%) was higher, followed by types III, II, I, and V (42.1%), while types VI, VII, and VIII were less frequent (5.9%). This was compatible with the trend reported in all the previously studied populations, except in Malaysia, where VC type I predominated (67.8%) [24], and India, Turkey, and Cyprus, where there were contradictory results related to the predominance of type IV or I [18, 19, 30–32].

The morphology of MFP in this study was similar according in the number of root and canal configuration between the two subsamples selected by quadrant. Although this result does not represent a direct comparison of right and left MFP, there was consensus in all the studies reporting this result, finding bilateral symmetry in the number of roots [3, 15–19, 23, 24, 26–32] and VC types [3, 16–18, 21, 23, 26, 30–32] in the same individual. Uniform formation is relevant for the bilateral treatment of canals in the dental office [18].

We found sex to be associated with the number of roots [15, 20, 21, 23, 27, 28, 30] and types of canals [15, 21, 23, 30, 32] similar to several previous populations but contrary to the results of other studies [3, 17, 18, 24, 28, 29, 31]. Most reports agree with our results of male sex being associated with double roots [15, 20, 21, 27, 28, 30] and VC type IV [15, 21, 23, 28, 30, 32], and women with single roots [15, 20, 21, 27, 28, 30] and types I [15, 21, 23, 28, 30] and II [23, 32]. Only one German study described an opposite association regarding the number of roots according to sex [23]. It is believed that growth hormones, combined with the action of sex steroids on osteoblastic cells, could be related to the dimensions of dental roots [36].

In relation to comparisons between root morphology versus canals, numerous studies have associated double roots with VC type IV in > 58% of cases [15–17, 20, 28, 29, 32], and single roots with type IV in 22%–59% [17, 20, 24, 28, 32], type II in 22%–53% [15–17, 19, 20, 24, 29, 32], type I in 20%–55% [15, 28, 29], and type III in 23% [16]. We also found double roots to be related to VC type IV (86%) and single roots to types III (34%), II (32%), and I (26%). However, in populations in Malaysia and India, double roots were associated with VC type I in 100% of cases [19, 24].

Another result of this study was the association of older age with double roots and VC types II and IV, and younger age with single roots and VC type I. This was consistent with a previous study suggesting that dentin deposits with aging could alter the morphology by reducing the pulp chamber and narrowing the canals [21]. The quadrant, sex, and age variables evaluated in this study are necessary for accurate radiographic interpretation of variations in roots and canals.

Some limitations of the present study regarding the methodological comparisons were that multiple studies involved individuals under 18 years of age [3, 18, 20, 21, 24, 26, 28, 30–32], in whom there might still be a lack of internal radicular dentin formation, or older adults > 60 years old [16–18, 21, 22, 24, 28, 30–32], in whom there is a high probability of dentin deposit apposition [12]. Therefore, it is recommended that future studies divide the study populations into age groups for more homogeneous results.

## 5. Conclusions

Within the limitations of this study, it was concluded that the most common morphology of MFP in a sample of Peruvian adults was the presence of double roots with two separate canals from the chamber to the apex. Root and canal morphology varied according to age and sex, with double roots and VC type IV being associated with male sex and older age, and single root and types II and I with female sex and younger age. Roots and canals were symmetrical by quadrants and double roots were associated with VC type IV and single roots with types III, II, and I.

## Figures and Tables

**Table 1:** Results of CBCT articles regarding roots and canals morphology of maxillary first premolars using VC.

Author (year)	Demographics (*n*)					Characteristics CBCT			Number of roots (%)				Vertucci classification (%)									Associated variables				
	Country	Subjects	Female/male	Age	Teeth	Equipment	Voxel size (*μ*m)	Software	1	2	3	4	I	II	III	IV	V	VI	VII	VIII	Others	#Roots vs. sex	#Roots vs. quadrant	VC vs. sex	VC vs. quadrant	#Roots vs. VC
Yanqui et al. (this studio)	Peru	392	196/196	20–60	392	Vatech PaX-i3D	200	ITK-SNAP	37	59.9	3.1	—	9.7	11.7	12.5	52	8.2	0.8	2	3.1	—	Yes	No	Yes	No	Yes
De Lima et al. (2019) [15]	Brazil	268	311/185	NR	496	I-CAT	250	XoranCat	18.2	80.2	1.6	—	6.5	7.7	0.6	82.2	0.8	0.6	—	1.6	—	Yes	No	Yes	NR	Yes
Li et al. (2018) [16]	China	774	498/276	20–80	1387	Planmeca	75	Romerix	69.7	30	0.5	—	11.8	14.7	16.1	42.7	12.1	0.6	0.7	0.7	0.6	NR	No	NR	No	Yes
Wu et al. (2020) [17]	China	1268	288/360	18–65	1268	NewTom VG	150	QR-NNT	67.4	32	0.6	—	10.4	24.2	0.4	58.6	4.9	0.8	—	0.3	0.2	No	No	No	No	Yes
Felsypremila et al. (2015) [18]	India	246	NR/NR	15–70	418	Kodak 9500	200	KDIS Dental	48.8	51.2	—	—	15.9	26.1	—	58	—	—	—	—	—	NR	No	NR	No	NR
Kartik et al. (2022) [19]	India	200	NR/NR	NR	400	Planmeca	200	Romexis	29	71	—	—	74.8	15.5	1.8	6	2	—	—	—	—	NR	No	NR	NR	Yes
Asheghi et al. (2020) [20]	Iran	462	278/184	16–60	462	Planmeca	150	Romerix	50.2	48	1.8	—	8.9	15.1	0.9	71.6	1.3	0.2	—	2	—	Yes	NR	NR	NR	Yes
Aljawhar et al. (2023) [21]	Iraq	385	220/165	12–80	572	Gender	150	Invivo y RadiAnt	51.1	47.7	1.2	—	5.6	0.3	13.8	46.7	16.6	8.4	2.4	1.4	4.7	Yes	NR	Yes	No	NR
Kfir et al. (2020) [22]	Israel	400	308/92	20–73	400	Planmeca	150	Romerix	36	61	3	—	2	17	0.5	74	0.5	6	—	—	—	NR	NR	NR	NR	NR
Bürklein et al. (2017) [23]	Germany	700	315/385	NR	644	Planmeca	200	Romerix	36.4	62.4	1.2	—	3.9	6.5	—	68.5	7.9	12.3	0.2	2	—	Yes	No	Yes	No	NR
Pan et al. (2019) [24]	Malaysia	208	90/118	15–66	304	KaVo 3D eXam	250	EXam	67.8	31.9	0.3	—	76.3	23.8	15.5	22.3	5.3	13.6	2.4	—	—	No	No	NR	NR	Yes
Martins et al. (2018) [25] ^*∗*^	Portugal	670	243/427	51	714	Planmeca	200	Romerix	48.7	49.2	2.1	—	3.4	17.1	0.3	68.2	1	4.6	—	0.7	4.7	NR	NR	NR	NR	NR
Alqedairi et al. (2018) [3]	Saudi Arabia	707	396/311	16–71	334	Planmeca	90–300	Romerix	23.7	75.1	1.2	—	10.8	8.4	1.8	70.6	3.9	2.1	0.3	2.1	—	No	No	No	No	NR
Mashyakhy et al. (2021) [26]	Saudi Arabia	208	108/100	17–59	351	Accuitomo	250	Morita	40.7	57.5	1.7	—	3.7	6.8	7.7	63.8	14.8	0.3	—	—	2.8	NR	No	NR	No	NR
Al-Zubaidi et al. (2021) [27]	Saudi Arabia	250	125/125	18–60	500	Carestream	75	CS 3D	39.8	58.6	—	—	5.2	32.8	0.6	57.8	2.0	0	1.6	—	—	Yes	No	NR	NR	NR
Buchanan et al. (2020) [28]	South Africa	190	109/81	14–84	316	Planmeca	100–600	Romerix	44	54.1	1.9	—	8.9	7.3	4.7	71.8	2.2	2.2	—	2.8	—	Yes	No	No	NR	Yes
Abella et al. (2015) [29]	Spain	620	362/258	NR	430	Planmeca	75	Romerix	4	51.4	2.6	—	25.1	10.2	4.4	52.8	1.9	1.6	1.4	2.6	—	No	No	NR	NR	Yes
Ok et al. (2014) [30]	Turkey	849	421/428	14–84	1379	I-CAT	300	I-CAT	12.5	86.2	1.2	—	9.6	6.5	1.4	76.9	4.6	0.1	—	1	—	Yes	No	Yes	No	NR
Bulut et al. (2015) [31]	Turkey	404	199/205	15–77	511	Newtom 5G	250	NNT	49.5	49.5	1	—	62.6	34.1	0.8	1.9	0.6	—	—	—	—	No	No	No	No	NR
Celikten et al. (2016) [32]	Turkey-Cyprus	263	228/209	16–80	437	Newton 3G	NR	NNT	53.7	44.8	0.9	—	4.5	16.2	0.4	76.8	0.6	—	—	—	0.9	No	No	Yes	No	Yes

Abbreviations: CBCT, cone-beam computed tomography; NR, not reported; and VC, Vertucci's classification.

^*∗*^Only the sample from Portugal was selected.

#Variables were associated with a significance level at *P* < 0.05.

**Table 2:** Distribution of maxillary first premolars in a sample of Peruvian adults.

Variables	Sex		Total
	Male	Female	
Total^‡^	196 (100%)	196 (100%)	392 (100%)
Age (years)^†^	38.55 ± 11.54	38.89 ± 11.65	38.72 ± 11.54
Age groups^‡^			
20–29 years	49 (25%)	49 (25%)	98 (25%)
30–39 years	49 (25%)	49 (25%)	98 (25%)
40–49 years	49 (25%)	49 (25%)	98 (25%)
50–60 years	49 (25%)	49 (25%)	98 (25%)
Quadrant^‡^			
Left	98 (50%)	98 (50%)	196 (50%)
Right	98 (50%)	98 (50%)	196 (50%)

^‡^Variable measured in frequency and percentage.

^†^Variable measured with mean ± standard deviation.

**Table 3:** Root morphology in maxillary first premolars by sex, age, and quadrant in the study sample.

Variables	Number and type of roots			*P* value
	1 Straight	2 Buccal and palatal	3 Palatal, mesial, and distal	
Total	145 (37%)	235 (59.9%)	12 (3.1%)	<0.001 ^*∗∗∗*^
Age^‡^	—	—	—	0.004 ^*∗∗*^
Male	57 (39.3%)^b^	131 (55.7%)^a^	8 (66.7%)^a^	—
Female	88 (60.7%)^a^	104 (44.3%)^b^	4 (33.3%)^a^	—
Years^†^	35 (19)^b^	42 (18)^a^	36 (24)^ab^	0.002 ^*∗∗*^
Age groups^‡^	—	—	—	0.003 ^*∗∗*^
20–29 years	43 (29.7%)^ac^	51 (21.7%)^bc^	4 (33.3%)^a^	—
30–39 years	49 (33.8%)^a^	47 (20%)^c^	2 (16.7%)^a^	—
40–49 years	23 (15.9%)^b^	71 (30.2%)^a^	4 (33.3%)^a^	—
50–60 years	30 (20.7%)^bc^	66 (28.1%)^ab^	2 (16.7%)^a^	—
Quadrant^‡^	—	—	—	0.994
Left	72 (49.7%)^a^	118 (50.2%)^a^	6 (50%)^a^	—
Right	73 (50.3%)^a^	117 (49.8%)^a^	6 (50%)^a^	—

*Note*: Different superscript letters per columns represent significant differences in each variable. Different superscript letters per rows represent significant differences. Kruskal–Wallis test. Pearson's *χ*^2^ test with Bonferroni adjustment. *χ*^2^ test for a single sample.

^‡^Variable valued with frequency and percentage.

^†^Variable valued with median and interquartile range.

Significant at  ^*∗∗*^*P* < 0.01;  ^*∗∗∗*^*P* < 0.001.

**Table 4:** Canal morphology in maxillary first premolars by sex, age, quadrant, and number of roots in the study sample.

Variables	Vertucci classification type								*P* value
	I (1-1)	II (2-1)	III (1-2-1)	IV (2-2)	V (1-2)	VI (2-1-2)	VII (1-2-1-2)	VIII (3-3)	
Total	38 (9.7%)	46 (11.7%)	49 (12.5%)	204 (52%)	32 (8.2%)	3 (0.8%)	8 (2%)	12 (3.1%)	<0.001 ^*∗∗∗*^
Sex^‡^									
Male	17 (44.7%)^a^	12 (26.1%)^b^	23 (46.9%)^a^	112 (54.9%)^a^	19 (59.4%)^a^	2 (66.7%)^a^	3 (37.5%)^a^	8 (66.7%)^a^	0.022 ^*∗*^
Female	21 (55.3%)^a^	34 (73.9%)^a^	26 (53.1%)^a^	92 (45.1%)^b^	13 (40.6%)^a^	1 (33.3%)^a^	5 (62.5%)^a^	4 (33.3%)^a^	—
Age^†^	31.5 (11)^b^	43.5 (11)^a^	35 (17)^ab^	42 (18)^a^	42 (24)^ab^	20 (0)^ab^	34 (17)^ab^	36 (24)^ab^	<0.001 ^*∗∗∗*^
Age groups^‡^									
20–29 years	17 (44.7%)^a^	9 (19.6%)^a^	13 (26.5%)^a^	41 (20.1%)^b^	10 (31.3%)^a^	2 (66.7%)^a^	2 (25%)^a^	4 (33.3%)^a^	<0.001 ^*∗∗∗*^
30–39 years	16 (42.1%)^a^	10 (21.7%)^a^	18 (36.7%)^a^	44 (21.6%)^b^	3 (9.4%)^a^	1 (33.3%)^a^	4 (50%)^a^	2 (16.7%)^a^	—
40–49 years	2 (5.3%)^b^	8 (17.4%)^a^	12 (24.5%)^a^	60 (29.4%)^a^	11 (34.4%)^a^	0 (0%)	1 (12.5%)^a^	4 (33.3%)^a^	—
50–60 years	3 (7.9%)^b^	19 (41.3%)^a^	6 (12.2%)^a^	59 (28.9%)^a^	8 (25%)^a^	0 (0%)	1 (12.5%)^a^	2 (16.7%)^a^	—
Quadrant^‡^									
Left	17 (44.7%)^a^	23 (50%)^a^	26 (53.1%)^a^	103 (50.5%)^a^	15 (46.9%)^a^	2 (66.7%)^a^	4 (50%)^a^	6 (50%)^a^	0.993
Right	21 (55.3%)^a^	23 (50%)^a^	23 (46.9%)^a^	101 (49.5%)^a^	17 (53.1%)^a^	1 (33.3%)^a^	4 (50%)^a^	6 (50%)^a^	—
Number of roots									
Single	38 (26.2%)	46 (31.7%)	49 (33.8%)	1 (0.7%)	0 (0%)	3 (2.1%)	8 (5.5%)	0 (0%)	<0.001 ^*∗∗∗*^
Double	0 (0%)	0 (0%)	0 (0%)	203 (86.4%)	32 (13.6%)	0 (0%)	0 (0%)	0 (0%)	—
Triple	0 (0%)	0 (0%)	0 (0%)	0 (0%)	0 (0%)	0 (0%)	0 (0%)	12 (100%)	—

*Note*: Different superscript letters per row represent significant differences. Kruskal–Wallis test. Different superscript letters per column represent significant differences in each variable. Pearson *χ*^2^ test with Bonferroni adjustment. One sample *χ*^2^ test.

^‡^Variable valued with frequency and percentage.

^†^Variable valued with median and interquartile range.

Significant at  ^*∗*^*P* < 0.05;  ^*∗∗∗*^*P* < 0.001.

## Data Availability

The data are available upon request.

## Supporting Information

Supporting Information Table S1: STROBE statement—checklist of items that should be included in reports of cross-sectional studies.
